# Relevance of plasma lipoproteins and small metabolites in assessment of nutritional status among patients with severe injuries

**DOI:** 10.1016/j.jointm.2024.02.004

**Published:** 2024-04-10

**Authors:** Esmee A.H. Verheul, Suzan Dijkink, Pieta Krijnen, Aswin Verhoeven, Martin Giera, Roula Tsonaka, Jochem M. Hoogendoorn, Sesmu M. Arbous, Ron Peters, Inger B. Schipper

**Affiliations:** 1 Department of Trauma Surgery, Leiden University Medical Center, Leiden, The Netherlands; 2 Acute Care Network West Netherlands, Leiden, The Netherlands; 3 Center for Proteomics and Metabolomics, Leiden University Medical Center, Leiden, The Netherlands; 4 Department of Biomedical Data Sciences, Leiden University Medical Center, Leiden, The Netherlands; 5 Department of General Surgery, Haaglanden Medical Center, The Hague, The Netherlands; 6 Department of Intensive Care, Leiden University Medical Center, Leiden, The Netherlands; 7 Department of Intensive Care, Haaglanden Medical Center, The Hague, The Netherlands

**Keywords:** Trauma, Severely injured, Metabolites, Lipoproteins, Malnutrition, Nutritional status

## Abstract

**Background:**

This study aimed to identify plasma lipoproteins and small metabolites associated with high risk of malnutrition during intensive care unit (ICU) stay in patients with severe injuries.

**Methods:**

This observational prospective exploratory study was conducted at two level-1 trauma centers in the Netherlands. Adult patients (aged ≥18 years) who were admitted to the ICU for more than 48 h between July 2018 and April 2022 owing to severe injuries (polytrauma, as defined by Injury Severity Scores of ≥16) caused by blunt trauma were eligible for inclusion. Partial least squares discriminant analysis was used to analyze the relationship of 112 lipoprotein-related components and 23 small metabolites with the risk of malnutrition (modified Nutrition Risk in Critically Ill score). Malnutrition was diagnosed based on Subjective Global Assessment scores. The relationship of lipoprotein properties and small metabolite concentrations with malnutrition (during ICU admission) was evaluated using mixed effects logistic regression.

**Results:**

Overall, 51 patients were included. Lower (very) low-density lipoprotein ([V]LDL) (free) cholesterol and phospholipid levels, low particle number, and higher levels of LDL triglycerides were associated with a higher risk of malnutrition (variable importance in projection [VIP] value >1.5). Low levels of most (V)LDL and intermediate-density lipoprotein subfractions and high levels of high-density lipoprotein Apo-A1 were associated with the diagnosis of malnutrition (VIP value >1.5). Increased levels of dimethyl sulfone, trimethylamine N-oxide, creatinine, N, N-dimethylglycine, and pyruvic acid and decreased levels of creatine, methionine, and acetoacetic acid were also indicative of malnutrition (VIP value >1.5). Overall, 14 lipoproteins and 1 small metabolite were significantly associated with a high risk of malnutrition during ICU admission (*P* <0.05); however, the association did not persist after correcting the false discovery rate (*P*=0.35 for all).

**Conclusion:**

Increased triglyceride in several lipoprotein subfractions and decreased levels of other lipoprotein subfraction lipids and several small metabolites (involved in the homocysteine cycle, ketone body formation, and muscle metabolism) may be indicative of malnutrition risk. Following validation in larger cohorts, these indicators may guide institution of preventive nutritional measures in patients admitted to the ICU with severe injuries.

## Introduction

Malnutrition is a serious concern in hospitalized patients, as it is known to be associated with adverse events such as infections, prolonged hospital stay, impaired wound healing, and mortality.^[^[1], [2], [3]^]^ In this context, the American Society for Parenteral and Enteral Nutrition defines three types of malnutrition: (1) pure chronic starvation without inflammation, (2) malnutrition resulting from chronic disease or conditions that lead to sustained inflammation of a mild to moderate degree, and (3) malnutrition caused by acute disease or injury states with a marked inflammatory response.^[^^4^^]^ Patients with severe injuries predominantly experience acute malnutrition (type 3), as the body is in a hypermetabolic state after severe trauma.^[^^4^^,^^5^^]^ The incidence of in-hospital malnutrition ranges from 7 % to 76 % in these cases, depending upon the setting, population, and nutritional assessment tool used.^[^^5^^]^ Several tools are available for nutritional assessment; some help diagnose current malnutrition, while others screen for the risk of future malnutrition.^[^^6^^]^ Nevertheless, the objective in-hospital measurement of nutritional status in critically ill and severely injured patients remains a challenge owing to various factors. Mechanical ventilation often makes it difficult to obtain a dietary history, swelling and edema may hinder the accurate evaluation of muscle wasting, and the acute-phase response after inflammation or trauma may affect visceral protein (albumin and pre-albumin) concentrations.^[^[7], [8], [9]^]^ As most patients are healthy individuals prior to trauma, nutritional biomarkers may serve as better indicators of malnutrition in these patients than in those with other critical illnesses (as they may have more comorbidities).

Plasma lipoproteins and small metabolites (low-molecular-weight metabolites) are potentially useful laboratory parameters for the assessment of nutritional status and risk of malnutrition.^[^^10^^,^^11^^]^ Lipoproteins are involved in multiple processes such as cell membrane formation, energy storage, and fat-soluble vitamin transportation; they also serve as chemical messengers.^[^^12^^]^ High-density lipoproteins (HDLs) have been found to play an important role in the immune system, and are involved in the modulation of complement system activation, regulation of antigen-presenting functions in macrophages, and activation of B and T cells.^[^^13^^]^ Notably, HDL levels and function are altered in several auto-immune diseases including rheumatoid arthritis and multiple sclerosis, and during inflammatory responses.^[^^13^^]^ In addition, high levels of total and low-density lipoprotein (LDL) cholesterol have been found to be associated with increased cardiovascular mortality.^[^^14^^]^ In this context, small metabolites represent intermediate- or end-products of biochemical pathways, and are related to oxidative stress, muscle catabolism, and nucleotide synthesis.^[^^15^^]^ As malnutrition is also related to oxidative stress and muscle catabolism, the relation between plasma lipoprotein and small metabolite levels and malnutrition, and their potential value in the assessment of nutritional status, warrant investigation.^[^^16^^,^^17^^]^

Several small metabolites and vitamins appear to be associated with malnutrition in hospitalized patients. However, research pertaining to the value of lipoproteins and small metabolites in the assessment of nutritional status in severely injured patients is scarce.^[^^18^^]^ The available literature is limited by the paucity of potential biomarkers and measurement (of lipoproteins and small metabolites) at only one time point during hospital admission. This exploratory study aimed to identify the plasma lipoproteins and small metabolites that may be used to assess nutritional status in patients admitted to the intensive care unit (ICU) with severe injuries. We evaluated whether specific lipoproteins and small metabolites are associated with a high risk of malnutrition at admission in patients with severe injuries. We also assessed the relationship between plasma levels of these lipoproteins and small metabolites and the incidence and prevalence of malnutrition during ICU admission.

## Methods

This observational prospective exploratory study was conducted at two level-1 trauma centers in the Netherlands (Leiden University Medical Center and Haaglanden Medical Center Westeinde). The study was incorporated in the Malnutrition in Polytrauma Patients (MaPP) study, which was initiated in July 2018.^[^^19^^]^ The MaPP study and the present metabolomics substudy were approved by the local institutional review boards (protocol number: NL64016.058.17). Adult patients (aged ≥18 years) with severe injuries (having polytrauma, defined by Injury Severity Scores [ISS] of ≥16) caused by blunt trauma, who were admitted to the ICU of the two centers between July 2018 and April 2022, were eligible for inclusion. Only patients who were admitted to the ICU for more than 48 h and were not primarily managed in another hospital were included. Those with burn wounds and penetrating injuries were excluded. Informed consent was obtained from the patients or their legal representative on the day of ICU admission or as soon as possible. In cases where a legal representative had initially provided written informed consent, the patient was asked to confirm consent (if able to provide written informed consent later during the course of the study).

### Data collection

Patient data including those pertaining to the medical history, ISS, height and weight, and other clinical data recorded during hospital admission were obtained from the patient files and stored on the Castor EDC system.^[^^19^^,^^20^^]^ The type of nutritional support received (enteral nutrition, parenteral nutrition, or oral diet) was recorded daily. Data pertaining to the values of albumin and pre-albumin, as observed within 48 h of admission, were also collected.

### Study parameters

The Subjective Global Assessment (SGA) and modified Nutrition Risk in Critically Ill (mNUTRIC) scores were the main study parameters. We evaluated the association between these parameters and alterations in plasma lipoprotein and small metabolite levels in patients with severe injuries. In this context, the SGA and mNUTRIC scores represent the current nutritional status and future malnutrition risk, respectively.

#### SGA

The SGA score is a nutrition assessment tool, which can be used to diagnose malnutrition.^[^^6^^]^ The tool was developed to assess the nutritional status and predict clinical outcomes in surgical patients. It is therefore expected to offer better prediction outcomes in ICU patients than the Mini Nutritional Assessment (MNA), which was developed to assess nutritional status in an elderly population.^[^^6^^,^[21], [22], [23]^]^ The SGA score is based on weight change (past 2 weeks and past 6 months), changes in adequacy of dietary intake, gastrointestinal symptoms (less appetite, nausea, vomiting, and diarrhea), and functional capacity (dysfunction, bedridden, and difficulty with normal activities). The score also includes physical examination components including subcutaneous fat loss (around the eyes, triceps, and biceps) and muscle wasting (including those around the clavicle, knee, shoulder and quadriceps). The total scores range from 1 to 7, and are classified as follows: (1) well-nourished (scores 6–7), (2) mildly/moderately malnourished (scores 3–5), and (3) severely malnourished (scores 1–2). Groups B and C are often combined under one category (malnourished) in general practice.^[^^19^^]^ During this study, the SGA score was assessed at ICU admission, every 5 days during ICU admission, and at ICU discharge.

#### mNUTRIC

The mNUTRIC score is a nutrition screening tool, which is used to determine the risk of malnutrition.^[^^6^^]^ The tool identifies critically ill patients who are most likely to benefit from aggressive nutritional treatment and is the first risk assessment tool developed and validated specifically for critically ill patients.^[^^24^^,^^25^^]^ The score is calculated based on the Acute Physiology and Chronic Health Evaluation (APACHE) II score, Sequential Organ Failure Assessment (SOFA) score, age, burden of comorbidities, and number of days of hospital stay prior to ICU admission.^[^^24^^,^^26^^]^ Notably, the APACHE II score is used as a general measure of disease severity and the SOFA score provides information regarding the prognosis of critically ill patients.^[^^27^^,^^28^^]^ For this study, data pertaining to all parameters needed to calculate the mNUTRIC score were obtained on the day of admission. An mNUTRIC score of <5 and ≥5 was considered to indicate a low and high risk of malnutrition, respectively.

#### Lipoproteins and small metabolites

**Figure 1:**
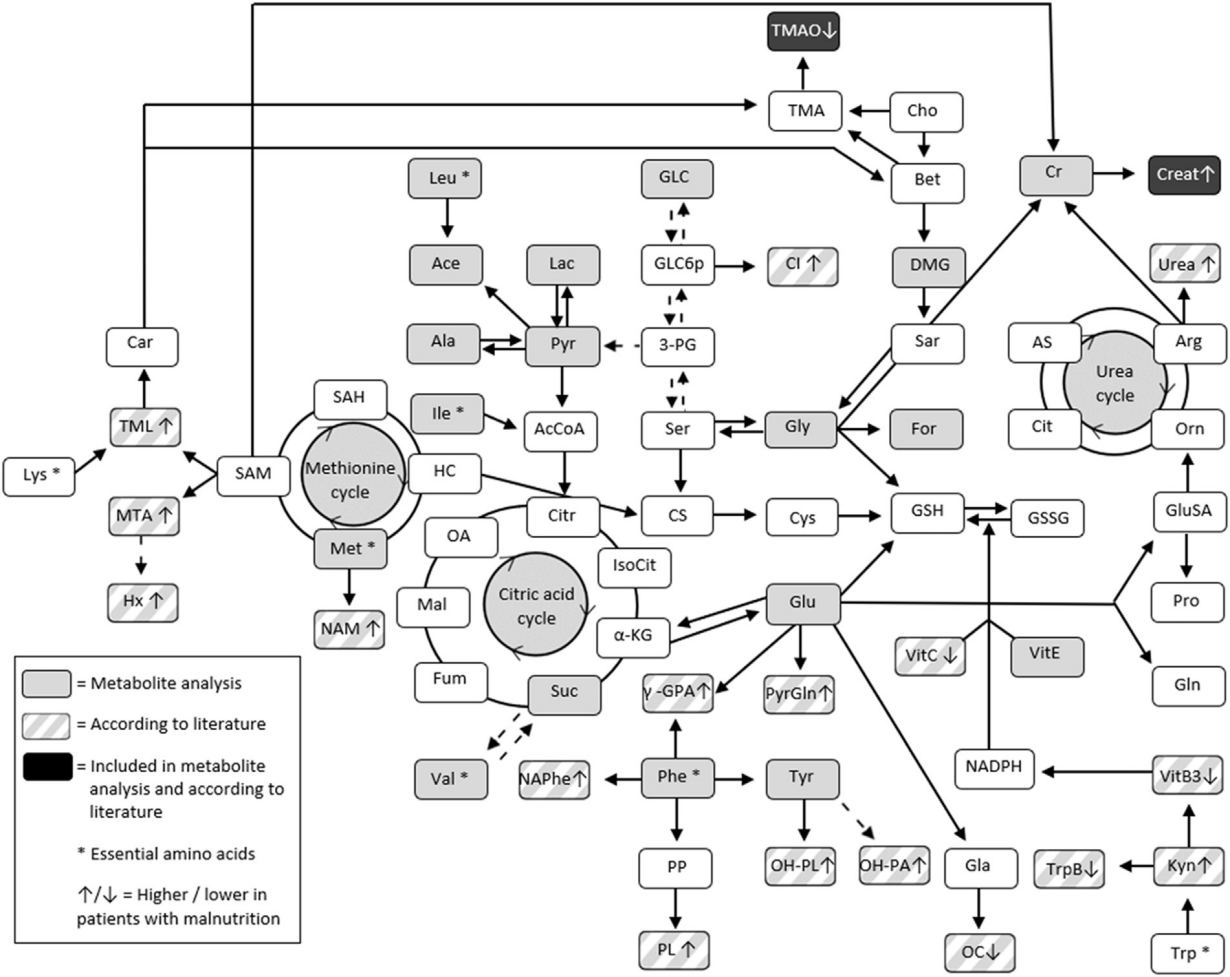
Small metabolites analyzed in this study.Associated metabolic cycles and relation with malnutrition (according to available literature). ^[18]^ Solid arrows signify single-step reactions and dotted arrows signify multiple steps. γ-GPA: γ-Glutamyl phenylalanine; α-KG: α-ketoglutarate; 3-PG: 3-phosphoglycerate; AcCoA: Acetyl-CoA; Ace: Acetic acid; Ala: Alanine; Arg: Arginine; AS: Argininosuccinate; Bet: Betaine; Car: Carnitine; Cho: Choline; CI: Chiro-inositol; Cit: Citrulline; Citr: Citrate; Cr: Creatine; Creat: Creatinine; CS: Cystathionine; Cys: Cysteine; DMG: N,N-Dimethylglycine; For: Formic acid; Fum: Fumarate; Gla: γ-carboxyglutamic acid; GLC: Glucose; GLC6p: Glucose-6-phosphate; Gln: Glutamine; Glu: Glutamic acid; GluSA: Glutamate-1-semialdehyde; Gly: Glycine; GSH: Reduced glutathione; GSSG: Glutathione disulfide; HC: Homocysteine; Hx: Hypoxanthine; Ile: Isoleucine; IsoCit: Isocitrate; Kyn: Kynurenine; Lac: Lactic acid; Leu: Leucine; Lys: Lysine; Mal: Malate; Met: Methionine; MTA: 5-Methylthioadenosine; NADPH: Nicotinamide-adenine-dinucleotidephosphate; NAM: N-acetylmethionine; NAPhe: N-acetylphenylalanine; OA: Oxaloacetate; OC: Osteocalcin; OH-PA: 4-hydroxyphenylacetate; OH-PL: 3-(4-hydroxyphenyl)lactate; Orn: Ornithine; Phe: Phenylalanine; PL: Phenyllactate; PP: Phenylpyruvate; Pro: Proline; Pyr: Pyruvic acid; PyrGln: Pyroglutamate; SAH: S-adenosylhomocysteine; SAM: S-adenosylmethionine; Sar: Sarcosine; Ser: Serine; Suc: Succinic acid; TMA: Trimethylamine; TMAO: Trimethylamine N-oxide; TML: N-6-trimethyllysine; Trp: Tryptophan; TrpB: Tryptophan Betaine; Tyr: Tyrosine; Val: Valine; VitB3: Vitamin B3 (nicotinamide); VitC: Vitamin C; VitE: Vitamin E.

In all cases, an additional tube of blood was drawn solely for the purpose of this research project. This was clearly explained in the study information that was provided to the patient and/or their relatives before obtaining informed consent and study inclusion. The plasma concentrations of a standard panel of lipoproteins and small metabolites were measured at the Center for Proteomics and Metabolomics of the Leiden University Medical Center using nuclear magnetic resonance (NMR).^[^^29^^]^ The NMR spectra of the plasma samples were acquired according to the protocols required by the Bruker In Vitro Diagnostics research platform (B.I. Methods); however, heparinized plasma samples were used instead of ethylenediaminetetraacetic acid plasma or serum samples (which are usually used). The lipoprotein and small metabolites were quantified automatically using the B.I.LISA and B.I.QUANT-PS web services.^[^^30^^]^ The full list of the 112 lipoproteins and 23 small metabolites that were analyzed, and the procedure of sample preparation for analysis, have been presented in supplementary materia. An overview of the small metabolites and related metabolic cycles evaluated in this study, and the relation between small metabolites associated with malnutrition (according to available literature), is shown in Figure 1.

### Statistical analysis

**Figure 2:**
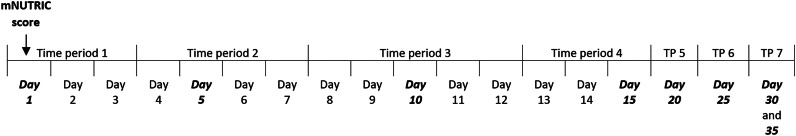
Clustering of daily metabolite analyses during the TPs. Bold and italicized: Day of nutritional assessment using the SGA tool. mNUTRIC: Modified Nutrition Risk in Critically Ill; SGA: Subjective Global Assessment; TP: Time period.

The sample size was not calculated, as this was an exploratory study. The collection of multiple samples per patient increased the study power.^[^^31^^]^ Statistical analyses were performed using IBM SPSS Statistics version 25 (IBM, Armonk, NY, USA),^[^^32^^]^ R version 4.2.2 (R Core Team, Vienna, Austria),^[^^33^^]^ and MetaboAnalyst 3.0 (Wishart Research Group, Alberta, Canada), a web-based metabolic data processing tool.^[^^34^^]^ *P*-values <0.05 were considered statistically significant. The baseline characteristics were compared between patients with low (mNUTRIC score <5) and high (mNUTRIC score ≥5) risk of malnutrition on admission.^[^^25^^]^ The Fisher's exact, independent samples T, and Mann–Whitney *U* tests were used for categorical variables, continuous variables with normal distribution, and continuous variables with non-normal distribution, respectively (using IBM SPSS Statistics). Outlier analysis was performed to detect the samples with extreme values; 4 samples with extremely high values (considered to reflect laboratory errors) were removed, as the values for lipoproteins and small metabolites differed considerably from those of other samples (>3 lipoprotein/small metabolite values with >6 standard deviation differences from the mean concentration). The samples were then divided into seven time periods (TPs), as shown in Figure 2. The average concentrations of the daily measurements within each TP were then calculated to assess the relationship with the corresponding SGA score. Lipoprotein and metabolic profiles were analyzed via three approaches, as described below.

#### Multivariate analysis was used to construct a lipoprotein and small metabolite-based model that could classify future malnutrition risk

The MetaboAnalyst tool was used to analyze the log-transformed lipoprotein and small metabolite levels during period 1 (days 1–3 of ICU admission; Figure 2). Partial least squares discriminant analysis (PLS-DA) was used to compare lipoproteins and small metabolites between patients with high and low risk of malnutrition, based on the mNUTRIC score on the day of ICU admission.^[^[35], [36], [37]^]^ In this context, PLS-DA is a multivariate dimensionality-reduction tool that has been recommended for use in metabolomics data analyses, in which the data sets often have considerably fewer samples than features.^[^^38^^]^ Cross-validation of the model resulted in a *Q*^2^ value, which provides an estimate of the predictive ability of the model. This value is determined by comparing the predictive and original data and calculating the sum of squared errors. The prediction error is then summed over all samples (predicted residual sum of squares). Good predictions have low predicted residual sum of squares or high *Q*^2^ values.^[^^34^^,^^39^^]^ A model with *Q*^2^=1 has perfect predictive accuracy, while that with *Q*^2^ <0 has no predictive power. Variable importance in projection (VIP) scores were calculated to assess the importance of each lipoprotein/small metabolite in the PLS model projection.^[^^40^^]^ VIP values of >1.5 were considered to be influential for discrimination between the groups with high and low risk of malnutrition.^[^^35^^]^ Multivariate receiver operating characteristic curve analysis was also performed based on PLS-DA, and an area under the receiver operating characteristic (AUROC) value was calculated for the model.^[^^36^^]^

#### A lipoprotein and small metabolite-based model was constructed using multivariate analysis to classify concomitant malnutrition

The log-transformed lipoprotein and small metabolite levels during period 2 (days 4–7 of ICU admission) were analyzed using MetaboAnalyst. PLS-DA was used to compare lipoproteins and small metabolites between malnourished patients, based on the SGA score on Day 5 of ICU admission. Notably, the majority of patients were well-nourished at the time of ICU admission (as this represented the pre-hospitalization nutritional status). However, many of the previously well-nourished patients were expected to have developed malnutrition during the second TP. The second TP was therefore selected to compare the malnourished and well-nourished groups.

#### Univariate analysis was performed to evaluate the association of lipoproteins and small metabolites with nutritional status

For the third analysis, a mixed effects logistic regression analysis (with repeated measures for each lipoprotein and small metabolite) was performed in R. The log-transformed concentrations of the mean lipoprotein and small metabolite levels were tested for their ability to indicate malnutrition (SGA score category B or C) during each TP (Figure 2). Baseline mixed effects logistic regression was performed for each lipoprotein and small metabolite, considering malnutrition as a binary outcome variable, TP as a fixed effect, and patient number as a random effect. A second mixed effects logistic regression was then fitted by adding the lipoprotein/small metabolite level (as the main effect) and an interaction term of the lipoprotein/small metabolite level with TP (as fixed effect) to the baseline model. The interaction term was added to allow for changes in the association between lipoprotein/small metabolite levels and malnutrition over time. The likelihood ratio test (LRT) was used to identify any association between malnutrition and each of the lipoprotein parameters and small metabolites over time.^[^^41^^]^ False discovery rate (FDR) correction was applied to the LRT *P*-values to correct for multiple testing, with a threshold of 0.05.^[^^42^^]^ The main effect of the lipoprotein/small metabolite and the interaction term have been presented for the models (of the lipoproteins/small metabolites) with significant *P*-values (at 5%), in addition to the β-coefficients for every unit increase and *P*-values for the TP. As the lipoprotein and small metabolite concentrations were log-transformed, the β-coefficients were multiplied by log_10_(1.10) and exponentiated to calculate the odds ratio (OR) for malnutrition (for a 10% increase in the lipoprotein/small metabolite concentration during each TP).

## Results

### Study population

Data from 51 patients in the MaPP study were included for analysis. Of these patients, 364 samples were collected in the seven time points according to Figure 2 (TP 1: 103 samples, TP 2: 129 samples, TP 3: 89 samples, TP 4: 28 samples, TP 5: 8 samples, TP 6: 5 samples, and TP 7: 2 samples). The median age of the cohort was 53.0 (interquartile range [IQR]: 32.0–64.0) years and 66.7% of patients were male (Table 1). Overall, 12 and 39 patients had a high (mNUTRIC score ≥5) and low (mNUTRIC score <5) risk of malnutrition on the day of ICU admission, respectively. These groups did not differ in terms of the body mass index, ISS, and Glasgow Coma Scale score at admission. Compared to one patient in the low-risk group, those with a high risk of malnutrition were significantly older (65.5 [IQR: 60.0–79.8] years] *vs*. 44.0 [IQR: 28.0–57.0] years; *P* <0.001), had a higher body weight (87.5 [IQR: 78.5–111.5] kg *vs.* 76.0 [IQR: 68.0–85.0] kg; *P*=0.030); three patients had a history of malignancy (*P*=0.036). The SOFA and APACHE II scores were significantly higher in the group with high mNUTRIC scores (*P* <0.001). The 30-day mortality rate was found to be 15.7% and no significant differences were found between the groups. The SGA scores at ICU admission did not differ between the groups. One patient in each group was found to be malnourished at admission, based on the SGA scores. All patients with severe injuries developed malnutrition after 20 days of ICU stay. The group with a high mNUTRIC score demonstrated significantly lower pre-albumin values than that with a low mNUTRIC score ([0.13±0.03] g/L *vs*. [0.17±0.06] g/L; *P*=0.015) (Table 1). Overall, 45 and 6 patients received enteral tube feeding and an oral diet, respectively. Tube feeding was started as soon as possible after ICU admission (between Day 1 and Day 3), depending on the need for preoperative fasting.

**Table 1:** Patient characteristics according to risk of malnutrition.

Characteristic	Low risk of malnutrition (mNUTRIC score <5) (*n*=39)	High risk of malnutrition (mNUTRIC score ≥5) (*n*=12)	*P*-value	Total (*n*=51)
Age (years)	44.0 (28.0–57.0)	65.5 (60.0–79.8)	<0.001	53.0 (32.0–64.0)
Male sex	25 (64.1)	9 (75.0)	0.728	34 (66.7)
Weight at admission (kg)	76.0 (68.0–85.0)	87.5 (78.5–111.5)	0.030	80.0 (69.0–90.0)
BMI at admission (kg/m^2^)	24.7 (23.1–27.1)	27.5 (23.0–33.2)	0.061	24.8 (23.1–28.7)
Obesity at admission (BMI ≥30 kg/m^2^)	4 (10.3)	5 (41.7)	0.024	9 (17.6)
SOFA score at admission	6.0±2.7	9.3±2.2	<0.001	6.8±2.9
APACHE II score at admission	14.4±5.6	23.1±4.3	<0.001	16.4±6.5
ISS	30.0 (25.0–41.0)	29.0 (25.3–37.0)	0.764	30.0 (25.0–38.0)
GCS score at admission			0.202	
13–15 (no/mild TBI)	11 (28.2)	2 (16.7)		13 (25.5)
9–12 (moderate TBI)	10 (25.6)	1 (8.3)		11 (21.6)
3–8 (severe TBI)	18 (46.15)	9 (75.0)		27 (52.9)
ICU admission days (days)*	7.5 (4.3–12.0)	10.0 (8.0–17.0)	0.092	8.0 (5.0–12.5)
Ventilator days (days)*	4.0 (1.3–9.8)	9.0 (3.5–11.0)	0.138	5.0 (2.0–10.0)
Malignancy	1 (2.6)	3 (25.0)	0.036	4 (7.8)
30-Day mortality	4 (10.3)	4 (33.3)	0.076	8 (15.7)
Malnourished according to SGA score				
D0	1/39 (2.6)	1/12 (8.3)	0.419	2/51 (3.9)
D5	11/32 (34.4)	4/12 (33.3)	1.000	15/44 (34.1)
D10	13/17 (76.5)	6/9 (66.7)	0.661	19/26 (73.1)
D15	8/10 (80.0)	2/2 (100.0)	1.000	10/12 (83.3)
D20	7/7 (100.0)	1/1 (100.0)	NA	8/8 (100.0)
Albumin (g/L)	33.3±5.9	30.7±4.8	0.169	32.7±5.7
Pre-albumin (g/L)	0.17±0.06	0.13±0.03	0.015	0.16±0.06

Data are expressed as *n* (%), mean ± standard deviation or median (interquartile range).

APACHE II: Acute Physiology and Chronic Health Evaluation; BMI: Body Mass Index; D0: Day of admission; D5: Day 5 of ICU admission; D10: Day 10 of ICU admission; D15: Day 15 of ICU admission; D20: Day 20 of ICU admission; GCS: Glasgow Coma Scale; ICU: Intensive Care Unit; ISS: Injury Severity Score; mNUTRIC: Modified Nutrition Risk in Critically Ill; NA: Not available; SGA: Subjective Global Assessment; SOFA: Sequential Organ Failure Assessment; TBI: Traumatic brain injury.

⁎ Patients who died during ICU admission were excluded.

### Predominant lipoproteins and small metabolites for the “risk of malnutrition” classifier

Lipoprotein and small metabolite values of two patients were not available for the first period of ICU admission. The data from the remaining 49 patients were therefore included for the PLS-DA; 10 and 39 of these patients had a high and low risk of malnutrition, respectively, as determined by the mNUTRIC scores. The mean levels of total triglycerides, total cholesterol, LDL cholesterol (LDL-C), and HDL cholesterol (HDL-C) were (159±153) mg/dL, (132±37) mg/dL, (59±26) mg/dL, and (45±14) mg/dL, respectively. The PLS-DA model was based on two partial least-squares components had a *Q*^2^ value of 0.04, indicating marginal predictive ability; the AUROC value of the model was 0.72.

**Figure 3:**
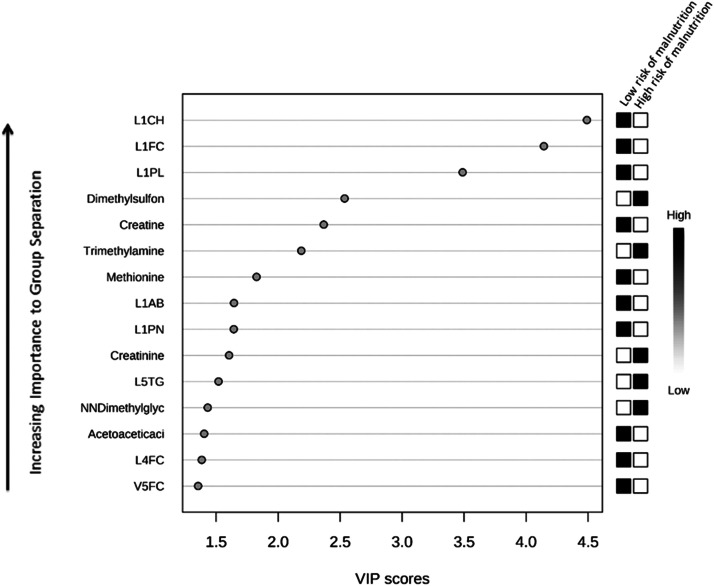
Risk of malnutrition biomarker identification using PLS-DA. PLS-DA was used to determine any relation between the risk of malnutrition (as defined by the mNUTRIC score) and the lipoprotein and small metabolite data. A VIP value was calculated to rank the top 15 lipoproteins and small metabolites according to their prognostic importance for the risk of malnutrition. The boxes on the right indicate the relative concentrations of the lipoprotein/small metabolite in the risk for malnutrition groups. For this analysis, the *Q*^2^ value is 0.04. L1AB: Apo-B Subfraction of LDL-1; L1CH: Cholesterol Subfraction of LDL-1; L1FC: Free Cholesterol Subfraction of LDL-1; L1PL: Phospholipids Subfraction of LDL-1; L1PN: LDL-1 Particle Number; L4FC: Free Cholesterol Subfraction of LDL-4; L5TG: Triglycerides Subfraction of LDL-5; mNUTRIC: Modified Nutrition Risk in Critically Ill; PLS-DA: Partial Least Squares Discriminant Analysis; V5FC: Free Cholesterol Subfraction of VLDL-5; VIP: Variable Importance in Projection.

The 15 lipoprotein parameters and small metabolites with the highest VIP values are shown in Figure 3. Specific subfractions of (V)LDL with low levels of (free) cholesterol and phospholipids, low particle number (Cholesterol Subfraction of LDL-1 [L1CH], Free Cholesterol Subfraction of LDL-1 [L1FC], Phospholipids Subfraction of LDL-1 [L1PL], Apo-B Subfraction of LDL-1 [L1AB], LDL-1 Particle Number [L1PN], Free Cholesterol Subfraction of LDL-4 [L4FC], and Free Cholesterol Subfraction of VLDL-5 [V5FC]), and high triglyceride levels of one of the LDL subfractions (L5TG), were indicative of a high risk of malnutrition. Increased levels of dimethyl sulfone, trimethylamine N-oxide (TMAO), creatinine, and N,N-dimethylglycine (DMG), and decreased levels of creatine, methionine, and acetoacetic acid, were also indicative of a high risk of malnutrition.

### Predominant lipoproteins and small metabolites for the “malnutrition on day 5” classifier

Data pertaining to lipoprotein and small metabolite levels were not available for seven patients during the second period of ICU admission. Therefore, 44 patients were included in the PLS-DA malnutrition model; 15 and 29 of these patients were malnourished and well-nourished, respectively, based on Day 5 SGA scores. The PLS-DA model was based on two partial least-squares components with a *Q*^2^ value of 0.09, indicating marginal predictive accuracy; the AUROC value of the model was 0.56.

**Figure 4:**
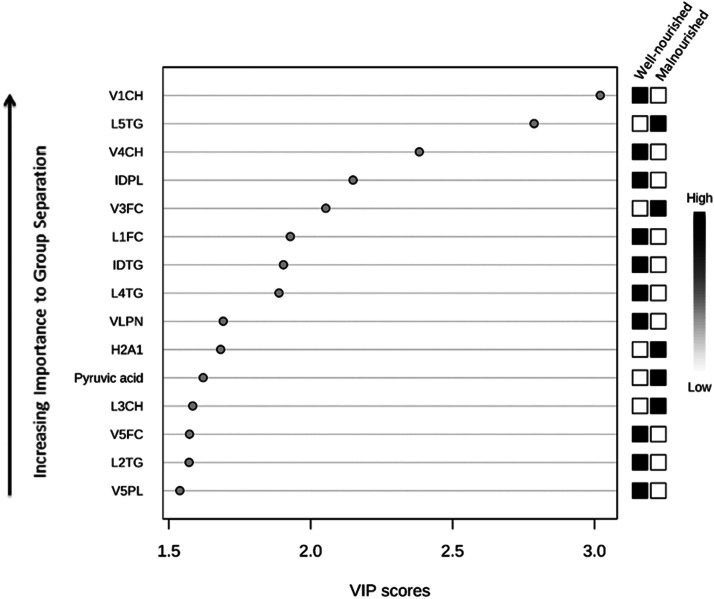
Malnutrition based on ICU day 5 biomarkers using PLS-DA. PLS-DA was used to relate the diagnosis of malnutrition on day 5 of ICU admission (defined by the SGA score) to the lipoprotein and small metabolite data. A VIP value was calculated to rank the top 15 lipoproteins and small metabolites according to their prognostic importance for the diagnosis of malnutrition. The boxes on the right indicate the relative concentrations of the lipoprotein/small metabolite in the nutritional status groups. For this analysis, the *Q*^2^ value was 0.09. H2A1: Apo-A1 Subfraction of HDL-2; ICU: Intensive Care Unit; IDPL: Lipoprotein Main Fractions, Phospholipids, IDL; IDTG: Lipoprotein Main Fractions, Triglycerides, IDL; L1FC: Free Cholesterol Subfraction of LDL-1; L2TG: Triglycerides Subfraction of LDL-2; L3CH: Cholesterol Subfraction of LDL-3; L4TG: Triglycerides Subfraction of LDL-4; L5TG: Triglycerides Subfractions of LDL-5; PLS-DA: Partial Least Squares Discriminant Analysis; SGA: Subjective Global Assessment; V1CH: Cholesterol Subfractions of VLDL-1; V3FC: Free Cholesterol Subfraction of VLDL-3; V4CH: Cholesterol Subfraction of VLDL-4; V5FC: Free Cholesterol Subfraction of VLDL-5; V5PL: Phospholipids Subfraction of VLDL-5; VIP, Variable Importance in Projection; VLPN: VLDL Particle Number.

The 15 lipoproteins and small metabolites with the highest VIP values are shown in Figure 4. Subfractions of (V)LDL and intermediate-density lipoprotein (IDL) with low levels of (free) cholesterol, particle number, triglyceride, and phospholipids (Cholesterol Subfraction of VLDL-1 [V1CH], Cholesterol Subfraction of VLDL-4 [V4CH], L1FC, L4TG, VLDL Particle Number [VLPN], V5FC, Triglycerides Subfraction of LDL-2 [L2TG], and Phospholipids Subfraction of VLDL-5 [V5PL]); (V)LDL subfractions with high levels of (free) cholesterol; and triglyceride subfractions (Free Cholesterol Subfraction of VLDL-3 [V3FC], L5TG, and Cholesterol Subfraction of LDL-3 [L3CH]) were indicative of malnutrition. High levels of Apo-A1 subfractions of HDL-2 (H2A1) and pyruvic acid were indicative of malnutrition.

### Lipoproteins and small metabolites associated with a change in nutritional status

Data from all 51 patients were included in the mixed effects logistic regression analysis. Among all 135 biomarkers, 14 lipoproteins and 1 small metabolite were found to significantly predict the risk of malnutrition during ICU admission (Table 2). However, the LRT *P*-value was no longer statistically significant after FDR correction (*P*=0.35 for all). Only the main effects of Apo-A2 Subfraction of HDL-4 (H4A2) and Triglycerides Subfraction of VLDL-1 (V1TG) were found to be significant (*P*=0.04 and *P*=0.03 respectively, Table 2). These lipoprotein subfractions had ORs of 0.77 and 0.89, respectively in period 1 (Table 3).

**Table 2:** Lipoproteins/small metabolites that were significant predictors of malnutrition according to the LRT.

Lipoprotein/small metabolite	Time period		Main effect		Interaction effect		*P*-value LRT	*P*-value LRT after FDR correction
	*P*-value	β-coefficient	*P*-value	β-coefficient	*P*-value	β-coefficient		
L5TG (mg/dL)	0.11	2.5±1.6	0.14	7.2±4.9	0.96	0.1±2.5	<0.01	0.35
L3FC (mg/dL)	0.39	1.0±1.2	0.70	−1.4±3.8	0.07	4.1±2.3	0.02	0.35
L3AB (mg/dL)	0.79	−0.5±1.9	0.73	−1.2±3.6	0.06	4.2±2.3	0.02	0.35
L3PN (nmol/L)	0.21	−5.8±4.6	0.73	−1.2±3.6	0.06	4.2±2.3	0.02	0.35
L4TG (mg/dL)	0.06	3.0±1.6	0.16	6.6±4.7	0.82	−0.5±2.2	0.02	0.35
L2FC (mg/dL)	0.93	−0.1±1.6	0.52	−1.9±3.0	0.07	4.5±2.5	0.03	0.35
L3PL (mg/dL)	0.66	0.7±1.6	0.77	−1.0±3.5	0.10	3.4±2.0	0.03	0.35
L6TG (mg/dL)	0.12	5.5±3.5	0.11	11.7±7.4	0.37	−3.6±4.0	0.03	0.35
L2PL (mg/dL)	0.82	−0.6±2.4	0.93	−0.3±3.6	0.16	3.8±2.7	0.03	0.35
Creatinine (mmol/L)	0.01	8.6±3.5	0.06	−11.8±6.2	0.06	4.2±2.2	0.04	0.35
L2CH (mg/dL)	0.94	−0.2±2.1	0.85	−0.5±2.9	0.16	2.9±2.0	0.04	0.35
L3TG (mg/dL)	0.11	3.4±2.2	0.13	9.7±6.4	0.65	−1.5±3.3	0.0463	0.35
H3A2(mg/dL)	0.22	−1.8±1.5	0.12	−6.4±4.1	0.01	6.7±2.6	0.0469	0.35
H4A2(mg/dL)	0.25	1.1±1.0	0.04	−6.2±3.0	0.10	1.9±1.1	0.0479	0.35
V1TG(mg/dL)	0.11	1.7±1.0	0.03	−2.7±1.3	0.13	1.1±0.7	0.0496	0.35

Mixed effects logistic regression analysis with repeated measures was used to evaluate the relationship between the nutritional status (defined by the SGA score) and the lipoprotein and small metabolite data. Overall, 14 lipoproteins and 1 small metabolite were found to be significant predictors according to the LRT, and therefore, appear to be associated with malnutrition over time. The *P*-value in the last column is after FDR correction. The β-coefficients for every unit increase and *P*-values of time period, the main effect of the lipoprotein/small metabolite, and the interaction term are shown (log10 transformed data).

FDR: False Discovery Rate; H3A2: Apo-A2 Subfraction of HDL-3; H4A2: Apo-A2 Subfraction of HDL-4; L2CH: Cholesterol Subfraction of LDL-2; L2FC:Free Cholesterol Subfraction of LDL-2; L2PL: Phospholipids Subfraction of LDL-2; L3AB: LDL Subfractions, Apo-B, LDL-3; L3FC: LDL Subfractions, Free Cholesterol, LDL-3; L3PL: Phospholipids Subfraction of LDL-3; L3PN: LDL-3 Particle Number, LDL-3 Particle Number; L3TG:Triglycerides Subfraction of LDL-3; L4TG: Triglycerides Subfraction of LDL-4; L5TG: LDL Subfractions, Triglycerides, LDL-5; L6TG: Triglycerides Subfraction of LDL-6; LRT: Likelihood Ratio Test; SGA: Subjective Global Assessment; V1TG: Triglycerides Subfraction of VLDL-1.

**Table 3:** ORs for malnutrition with a 10 % increase in the lipoprotein/small metabolite levels for every TP.

Lipoprotein/small metabolite	TP 1	TP 2	TP 3	TP 4	TP 5	TP 6	TP 7
L5TG(mg/dL)	1.35	1.36	1.36	1.37	1.38	1.39	1.39
L3FC(mg/dL)	0.94	1.12	1.33	1.57	1.87	2.21	2.62
L3AB(mg/dL)	0.95	1.13	1.35	1.61	1.92	2.29	2.73
L3PN(nmol/L)	0.95	1.13	1.35	1.61	1.92	2.29	2.73
L4TG(mg/dL)	1.31	1.29	1.26	1.23	1.21	1.19	1.16
L2FC(mg/dL)	0.92	1.11	1.34	1.62	1.95	2.35	2.83
L3PL(mg/dL)	0.96	1.10	1.27	1.46	1.67	1.92	2.21
L6TG(mg/dL)	1.63	1.40	1.21	1.04	0.89	0.77	0.66
L2PL(mg/dL)	0.99	1.15	1.35	1.57	1.84	2.15	2.51
Creatinine (mmol/L)	0.61	0.73	0.87	1.03	1.22	1.46	1.73
L2CH(mg/dL)	0.98	1.10	1.24	1.40	1.58	1.78	2.01
L3TG(mg/dL)	1.49	1.40	1.32	1.24	1.17	1.10	1.03
H3A2(mg/dL)	0.77	1.01	1.34	1.77	2.34	3.09	4.08
H4A2(mg/dL)	0.77	0.83	0.90	0.97	1.05	1.14	1.23
V1TG(mg/dL)	0.89	0.93	0.98	1.02	1.07	1.12	1.18

The ORs were calculated for the models of lipoproteins and small metabolites with a significant LRT *P*-value. As the lipoprotein and small metabolite concentrations were log-transformed, the β-coefficients were multiplied by log10(1.10) and exponentiated to calculate the ORs for malnutrition with a 10 % increase in the lipoprotein/small metabolite levels for every TP.

H3A2: Apo-A2 Subfraction of HDL-3; H4A2: Apo-A2 Subfraction of HDL-4; L2CH: Cholesterol Subfraction of LDL-2; L2FC:Free Cholesterol Subfraction of LDL-2; L2PL: Phospholipids Subfraction of LDL-2; L3AB: LDL Subfractions, Apo-B, LDL-3; L3FC: LDL Subfractions, Free Cholesterol, LDL-3; L3PL: Phospholipids Subfraction of LDL-3; L3PN: LDL-3 Particle Number, LDL-3 Particle Number; L3TG:Triglycerides Subfraction of LDL-3; L4TG: Triglycerides Subfraction of LDL-4; L5TG: LDL Subfractions, Triglycerides, LDL-5; L6TG: Triglycerides Subfraction of LDL-6; LRT: Likelihood Ratio Test; OR, Odds ratio; SGA: Subjective Global Assessment; TP: Time period,V1TG, Triglycerides Subfraction of VLDL-1.

## Discussion

The findings from this study imply that at ICU admission, LDL subfractions with increased levels of triglycerides and (V)LDL subfractions with decreased levels of (free) cholesterol, phospholipids, and decreased particle numbers were associated with a high risk of malnutrition. Subfractions of (V)LDL and IDL with decreased levels of (free) cholesterol, triglyceride, and phospholipids, lower particle number, and increased levels of (V)LDL (free) cholesterol, (V)LDL triglyceride subfractions, and HDL Apo-A1 were indicative of malnutrition on Day 5. HDL Apo-A2 and (V)LDL-free cholesterol may have been associated with malnutrition during ICU admission. Additionally, increased levels of dimethyl sulfone, TMAO, creatinine, and DMG, and decreased levels of creatine, methionine, and acetoacetic acid were found to be related to a high risk of malnutrition at ICU admission. Increased levels of pyruvic acid were indicative of malnutrition on Day 5 of ICU admission.

### Lipoproteins

Numerous lipoprotein subfraction parameters were investigated in terms of their relation with the nutritional status and risk of malnutrition; these included (V)LDL particles which transport cholesterol to the peripheral tissues and HDL particles which take excess cholesterol and return it to the liver for excretion.^[^^43^^,^^44^^]^ In their meta-analysis, Zhang et al.^[^^11^^]^ evaluated the association between blood biomarkers (including LDL and HDL) and the nutritional status (assessed using the MNA) in elderly patients. They found no significant difference in HDL between the three groups (based on the MNA score); they also found the LDL levels to be significantly lower in the malnourished patients. In this context, the relationship between lipoprotein levels and malnutrition may be partly explained by the production of cytokines. Malnutrition is found to be related to an increase in several cytokines including interferon-γ, interleukins 2 and 4, and tumor necrosis factor-α (TNF-α).^[^^45^^]^ Notably, TNF-α is known to increase fatty acid levels by increasing both fatty acid synthesis and adipose tissue lipolysis. The fatty acids are re-esterified into triglycerides and released into the circulation as (V)LDL.^[^^46^^]^ Cytokines decrease both LDL-C and HDL-C levels by inhibiting cholesterol synthesis and decreasing cholesterol secretion.^[^^46^^]^ These findings are in concordance with ours. In our study, decreased levels of (free) cholesterol and phospholipids in subfractions of (V)LDL and decreased particle numbers during the first 3 days of admission (period 1) were associated with a high risk of malnutrition. Increased levels of LDL triglycerides during this period were also related to a high risk of malnutrition (Figure 3). In addition, several (V)LDL related parameters were found to be indicative of malnutrition on Day 5. Among the 14 lipoprotein subfraction variables with the highest VIP value, the majority included (V)LDL and IDL particles; these were indicative of malnutrition when decreased during period 2 (Figure 4). Interestingly, a study that compared lipid profiles between patients with sepsis and trauma found no significant difference in terms of LDL-C and triglyceride levels. Although HDL-C levels were markedly low during sepsis, no change was observed in the early phase of trauma (relative to standard HDL concentrations).^[^^47^^]^ In this context, several studies found LDL-C, HDL-C, and total cholesterol levels to be significantly lower and triglyceride levels to be significantly higher in patients suffering from severe acute respiratory syndrome coronavirus 2 (compared to control subjects).^[^[48], [49], [50]^]^ This dyslipidemia may have been caused by the production of cytokines; however, it may also be attributed to liver damage and increased degradation by free radicals consequent to the infection.^[^^48^^]^ In another study on patients with sepsis, the levels of LDL-C, HDL-C, and total cholesterol were decreased and those of triglyceride were increased at admission.^[^^51^^]^

In our univariate generalized linear mixed models, 14 lipoprotein parameters and 1 small metabolite were found to be significantly related to the nutritional status during ICU admission. However, this association did not remain significant after FDR correction (Table 2). The identified lipoprotein parameters and metabolites may have therefore demonstrated significance due to the large number of parameters (135 in total) analyzed, and may represent false positives. This factor needs to be considered when interpreting the results. Table 2 shows the β-coefficients and *P*-values of the TPs, individual factors (main effect) of the 14 lipoproteins and small metabolites, and interaction terms. Only the main effects of HDL Apo-A2 and (V)LDL triglycerides were found to be significantly related to the nutritional status (H4A2, V1TG), with a β-coefficient of −6.23 and −2.75, respectively. This indicates that a 1-unit increase in the log-transformed values of these lipoprotein subfractions during period 1 reduced the log-odds of malnutrition by 6.23- or 2.75-fold, respectively. Table 3 shows the ORs for malnutrition for a 10 % increase in the levels of these 14 lipoproteins and 1 small metabolite for each TP. The odds of malnutrition during period 1 was lowered by 23 % and 11 % for every 10 % increase in H4A2 and V1TG, respectively (OR=0.77 and 0.89, respectively). An increase in H4A2 and V1TG during the first four or three TPs, respectively, indicated a decrease in the risk of malnutrition. After these periods, an increase in levels was more likely to be related to a decrease in the risk of malnutrition. Notably, a decrease in Apo-A2 is also seen in cases of inflammation and infection.^[^^46^^]^ The main effects of the other lipoproteins were not significantly related to malnutrition; this may be attributed to the lack of power. Interestingly, the ORs for LDL (free) cholesterol, Apo-B, particle number, and phospholipid levels (Free Cholesterol Subfraction OF LDL-3 [L3FC], Apo-B Subfraction of LDL-3 [L3AB], LDL-3 Particle Number [L3PN], Free Cholesterol Subfraction of LDL-2 [L2FC], Phospholipids Subfraction of LDL-3[L3PL], Phospholipids Subfraction of LDL-2 [L2PL], and Cholesterol Subfraction of LDL-2 [L2CH]) included all negative values during period 1. This indicated that an increase in these LDL subfractions may be related to a decreased risk of malnutrition; however, all values were not significant (Table 3). An increase in the levels of these LDL subfractions from period 2 may be attributed to malnutrition. Interestingly, a similar trend has been seen in septic patients. In a study, the LDL-C and HDL-C levels were found to have decreased during the first 3 days of ICU admission. Although the levels were higher at ICU discharge than at Day 3,the levels measured prior to hospitalization were not attained.^[^^51^^]^ These changes in lipoprotein levels appeared to be restored after 7 days of admission.^[^^52^^]^ In our cohort, increased levels of L5TG were found to be related to a high risk of malnutrition (Figure 3). Increasing levels of LDL triglyceride may have also been related to malnutrition during ICU admission; however, the association was not significant (Table 2). As mentioned before, this may be explained by the increased production of TNF-α, which causes an increase in triglyceride levels.^[^^46^^]^

### Small metabolites

In patients with severe injuries or illness, large quantities of muscle proteins are broken down owing to the release of stress-hormones and cytokines. Alanine is transported to the liver and converted into pyruvate, and amino-acids are transformed into glucose and positive acute-phase proteins (such as fibrinogen and C-reactive protein) via gluconeogenesis.^[^^53^^]^ In our study, decreased levels of the amino acid methionine were found to be related to a high risk of malnutrition (Figure 3). In this context, methionine is a precursor of homocysteine (HC), and hyperhomocysteinemia is known to be a risk factor for cardiovascular disease, cognitive impairment, and Alzheimer's disease. Deficiencies in micronutrients, such as vitamin B12 and folate, are known to influence HC concentrations. HC can be remethylated by either the methionine synthase or betaine-HC S-methyltransferase pathways. DMG is a product of the latter; an increase in its levels was found to be related to an increased risk of malnutrition (Figure 3).^[^^54^^,^^55^^]^ N-acetylmethionine is also related to the HC cycle, and increased levels have already been found to be associated with malnutrition in critically ill patients.^[^^10^^]^ Notably, the liver begins to transform fatty acids into ketone bodies during continued fasting; the latter can be used by the brain as the main energy source.^[^^56^^,^^57^^]^ Increased levels of acetoacetic acid, one of the ketone bodies, have been found to be related to an increased risk of malnutrition (Figure 3).

Increased levels of TMAO have also been found to be related to a high risk of malnutrition during the first few days of ICU admission (Figure 3). TMAO is a pro-inflammatory metabolite that originates from the bacterial metabolism of choline-rich foods. Elevated TMAO levels have been found to be associated with coronary artery disease, chronic kidney disease, and chronic obstructive pulmonary disease. In their study, Chou et al.^[^^58^^]^ found that decreased levels of TMAO were associated with acute and chronic malnutrition in septic patients. They, however, observed that antibiotic treatment and liver dysfunction were also significantly associated with a decrease in TMAO levels; this may explain the difference in our results.

Increased levels of creatinine, an endogenous product of muscle metabolism, have been found to be related to malnutrition.^[^^59^^]^ In our study, increased creatinine levels were found to be associated with a high risk of malnutrition (Figure 3). An increase in creatinine concentrations may therefore be related to malnutrition; however, the association was not significant (Table 2). Creatine is the precursor to serum creatinine and is synthesized in the liver. Decreased serum creatine levels on ICU admission were found to be related to a high risk of malnutrition (Figure 3). This may be attributed to decreased liver function or muscle mass.^[^^59^^,^^60^^]^

### Limitations

As this was an exploratory pilot study, the time available for patient recruitment was limited; this led to a relatively small sample size. Therefore, it was not possible to perform subanalyses based on certain variables (for example, gender, age, or trauma site). This issue was addressed to a certain extent by obtaining multiple samples per patient; this provided the added opportunity to track the course of malnutrition over time. Additionally, this exploratory study was conducted as part of a larger observational prospective study and according to the routines in daily practice. Blood samples were therefore not obtained on the exact day of assessment of nutritional status; average values of the plasma levels obtained around the day of assessment were used instead. Subtle fluctuations over time may have therefore been overlooked. However, the fluctuations were expected to be minimal and probably had negligible influence. In this context, changes in lipoprotein and small metabolite levels are not the only factors related to the deterioration of nutritional status. Inflammation, oxidative stress, medication, and comorbidities also play a role in many metabolic processes within the body and influence the nutritional parameters. The lack of a gold standard for assessment of (the risk of) malnutrition represents a major limitation for all studies pertaining to the condition. We used the SGA and mNUTRIC scores, as those are validated for ICU patients and have been proven to have the highest predictive value for outcomes. The SGA score by itself is known to be an approximate measure, as the difference between an SGA score of 5 (malnourished) or 6 (well-nourished) can be considerably minimal. The models were therefore trained using imperfect data, which limited model performance. The SGA scores were verified by one investigator at the end of data collection to increase reliability and reduce interobserver variability. Additionally, no difference was made in terms of the severity of malnutrition, as SGA scores of 1 to 5 are all considered to indicate malnutrition. Unfortunately, the number of included patients with severe malnutrition (SGA ≤2) was inadequate for performing subanalyses. The lipid intake (including enteral and oral feeding) and propofol infusion were not considered during the analyses; this represents another limitation. In addition, VIP values were used to identify the lipoproteins and small metabolites that could influence discrimination between groups in the PLS-DA regression model; however, the model itself demonstrated marginal predictive ability for malnutrition. More patients need to be included in the analysis in order to increase the predictive power of the model. Additionally, NMR is considered a considerably expensive method for analysis; this represents a challenge to the incorporation of metabolite and lipoprotein analyses into everyday clinical practice. An alternative includes Lipoprint® (Mayo Clinic and Foundation, MN, USA), which is a relatively rapid system compared with most gel electrophoresis methods and is less expensive.^[^^61^^]^ Lastly, the VIP values only reflect the importance of each variable in the projection used in this specific PLS model. Therefore, these results need to be validated using larger sample sizes; this will allow the calculation of cut-off values for normal ranges of lipoproteins and small metabolites prior to their use in clinical practice. The incorporation of lipoprotein and small metabolite analysis in routine care will represent a major step forward in providing personalized medicine, which considers individual differences in metabolism.

## Conclusions

The findings from this study suggest that increased triglyceride levels in several plasma lipoprotein (sub)fractions and decreased levels of other lipoprotein subfractions may be associated with a high risk of malnutrition in patients with severe injuries; they may also be associated with a decrease in nutritional status during ICU admission. Additionally, small metabolites involved in the HC cycle, ketone body formation, and muscle metabolism may be indicative of (the risk of) malnutrition. Following validation of our findings in studies with larger sample sizes, the identified biomarkers may be used as indicators for an institution of preventive nutritional measures in patients admitted to the ICU with severe injuries. As malnutrition is not the only process that influences metabolic patterns, further research is needed to investigate the value of lipoproteins and small metabolites in diagnosing malnutrition and assessing the risk of developing the condition.

## CRediT authorship contribution statement

**Esmee A.H. Verheul:** Writing – review & editing, Writing – original draft, Visualization, Methodology, Investigation, Funding acquisition, Formal analysis, Data curation, Conceptualization. **Suzan Dijkink:** Writing – review & editing, Supervision, Methodology, Investigation, Funding acquisition, Formal analysis, Data curation, Conceptualization. **Pieta Krijnen:** Writing – review & editing, Supervision, Methodology, Investigation, Funding acquisition, Formal analysis, Conceptualization. **Aswin Verhoeven:** Writing – review & editing, Visualization, Software, Resources, Formal analysis, Data curation. **Martin Giera:** Writing – review & editing, Visualization, Software, Resources, Formal analysis, Data curation. **Roula Tsonaka:** Writing – review & editing, Formal analysis. **Jochem M. Hoogendoorn:** Writing – review & editing, Data curation. **Sesmu M. Arbous:** Writing – review & editing, Data curation. **Ron Peters:** Writing – review & editing, Data curation. **Inger B. Schipper:** Writing – review & editing, Supervision, Methodology, Investigation, Funding acquisition, Formal analysis, Data curation, Conceptualization.

## Appendix Supplementary materials

Image, application 1
