# Supplementary material for: Relevance of plasma lipoproteins and small metabolites in assessment of nutritional status among patients with severe injuries
Source: J Intensive Med. 2024 Apr 10;4(4):496–507. doi: 10.1016/j.jointm.2024.02.004 (PMC11411433; doi:10.1016/j.jointm.2024.02.004)
Supplement: Supplementary file 1 [file mmc1.docx]

**Appendix 1**

*Small metabolites*

- Ethanol
- Trimethylamine-N-oxide (TMAO)
- Alanine
- Creatine
- Creatinine
- Glutamic acid
- Glycine
- Histidine
- Isoleucine
- Leucine
- Methionine
- N,N-Dimethylglycine
- Phenylalanine
- Tyrosine
- Valine
- Acetic acid
- Formic acid
- Lactic acid
- Succinic acid
- Acetoacetic acid
- Pyruvic acid
- Glucose
- Dimethyl sulfone

*Lipoproteins*

- Apolipoproteins -A1 (total and HDL (total & 1-4))
- Apolipoproteins -A2 (total and HDL (total & 1-4))
- Apolipoproteins -B (total, VLDL, IDL (total & 1-6), LDL)
- Particle number (total, VLDL, IDL, LDL, LDL 1-6)
- Triglycerides (total, VLDL (total & 1-6), IDL, LDL (total & 1-6), HDL (total & 1-4))
- Cholesterol (total, VLDL (total & 1-6), IDL, LDL (total & 1-6), HDL (total & 1-4))
- Free cholesterol (VLDL (total & 1-6), IDL, LDL (total & 1-6), HDL (total & 1-4))
- Phospholipids (VLDL (total & 1-6), IDL, LDL (total & 1-6), HDL (total & 1-4))

*Sample preparation Nuclear Magnetic Resonance (NMR) serum measurements*

The sample preparation was performed according to the requirements of the Bruker B.I.LISA lipoprotein analysis protocol, except for the use of heparin plasma instead of EDTA plasma or serum. The plasma samples were thawed at room temperature. Immediately after thawing the samples were homogenized by inverting the tubes 10 times. Next, 500 μl of serum was manually transferred to a Ritter 96 deepwell plate. A Gilson 215 liquid handler robot was used to mix 300 µL of plasma with 300 µLof 75 mM disodium phosphate buffer in H2O/D2O (80/20) with a pH of 7.4 containing 6.15 mM NaN3 and 4.64 mM sodium 3-[trimethylsilyl] d4-propionate (Cambridge Isotope Laboratories). Using a modified second Gilson 215 liquid handler, 565 μl of each sample was transferred into 5 mm Bruker SampleJet NMR tubes. Subsequently the tubes were closed by POM ball insertion and transferred to the SampleJet autosampler where they were kept at 6°C while queued for acquisition.

*NMR experiments and processing*

All proton nuclear magnetic resonance (1H-NMR) experiments were acquired on a 600 MHz Bruker Avance Neo spectrometer (Bruker Corporation, Billerica, USA) equipped with a 5 mm triple resonance inverse (TCI) cryogenic probe head with Z-gradient system and automatic tuning and matching.

The NMR spectra were acquired following the Bruker B.I.Methods protocol. A standard 5 mm sample of 99.8% methanol-d4 (Bruker) was used for temperature calibration (Findeisen, M., Brand, T. & Berger, S. A. Magnetic Resonance in Chemistry 45, 175–178, 2007) before the measurements. A standard 5 mm QuantRefC sample (Bruker) was measured as the quantification reference and for quality control. All experiments were recorded at 310 K. The duration of the π/2 pulses were automatically calibrated for each individual sample using a homonuclear-gated nutation experiment (Wu, P. S. C. & Otting, G. Journal of Magnetic Resonance 176, 115–119, 2005) on the locked and shimmed samples after automatic tuning and matching of the probe head. For water suppression, presaturation of the water resonance with an effective field of γB1 = 25 Hz was applied during the relaxation delay and the mixing time of the NOESY1D experiment (Price, W. S. Annual Reports on NMR Spectroscopy 38, 289–354, 1999).

The NOESY1D experiment was recorded using the first increment of a NOESY pulse sequence (Kumar, A., Ernst, R. R. & Wüthrich, K. Biochemical and Biophysical Research Communications 95, 1–6, 1980) with a relaxation delay of 4 s and a mixing time of 10 ms. 32 scans of 98,304 points covering a sweepwidth of 17,857 Hz were recorded after applying 4 dummy scans.

The lipoprotein values were extracted from the NOESY1D serum spectra by submitting the data to the commercial Bruker IVDr Lipoprotein Subclass Analysis (B.I.-LISA) platform.
